# ARID1A and Its Impact Across the Hallmarks of Cancer

**DOI:** 10.3390/ijms26104644

**Published:** 2025-05-13

**Authors:** Bridger Kearns, Andralyn McKell, Isaac Steveson, Peyton Worley, Braeden Barton, Jordan Bennett, DeLaney Anderson, Jacob Harris, James Christensen, Jared J. Barrott

**Affiliations:** 1 Department of Cell Biology & Physiology, Brigham Young University, Provo, UT 84602, USA; bkearns9@student.byu.edu (B.K.); andramc@student.byu.edu (A.M.); isaacjs7@student.byu.edu (I.S.); worleyp@student.byu.edu (P.W.); bartobra@student.byu.edu (B.B.); jmb472@student.byu.edu (J.B.); dgrace18@student.byu.edu (D.A.); jacobh23@student.byu.edu (J.H.); jsc810@student.byu.edu (J.C.); 2 Simmons Center for Cancer Research, Brigham Young University, Provo, UT 84602, USA

**Keywords:** ARID1A, genomic instability, immune evasion, proliferation, dedifferentiation, synthetic lethality

## Abstract

ARID1A, a subunit of the SWI/SNF chromatin remodeling complex, has emerged as a pivotal tumor suppressor altered in a broad range of human malignancies. Its frequent inactivation across diverse cancer types has revealed pleiotropic roles that intersect multiple Hallmarks of Cancer. In this review, we integrate current knowledge on how ARID1A loss influences cellular processes including proliferative signaling, resistance to cell death, genomic instability, metabolic reprogramming, immune evasion, and more. We discuss the context-specific consequences of ARID1A deficiency, its cooperation with other oncogenic events, and its implications for therapeutic vulnerability—particularly in the realm of synthetic lethality and immune modulation. By mapping ARID1A’s functional impact onto the established hallmarks framework, we highlight its centrality in cancer biology and underscore opportunities for biomarker-driven strategies and targeted interventions. Understanding ARID1A’s multifaceted roles offers a compelling lens through which to explore chromatin dysregulation in cancer and guide translational advances.

## 1. Introduction

**Figure 1:**
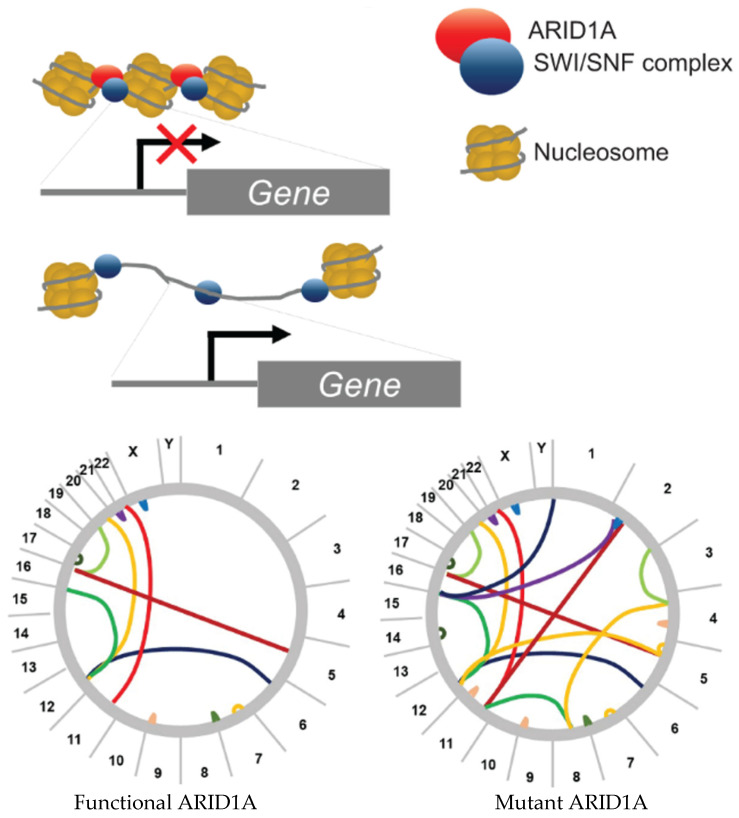
Chromatin remodeling and putative mechanism explaining how ARID1A loss results in enhanced genomic instability in cancer. The bottom circos plots demonstrate the theoretical genomic instability that can ensue once ARID1A is lost or mutated. The lines represent translocations between chromosomes due to double-strand breaks.

ARID1A (BAF250a or SMARCF1) is a member of the SWI/SNF (BAF) protein complex and is responsible for DNA binding. This protein complex regulates chromatin structure by binding nucleosomes and facilitating the unwinding or compaction of chromatin. Dysregulation of the complex can open the chromatin to aberrant gene transcription and chromosomal rearrangements (Figure 1) [1]. However, due to the multiple protein binding domains found throughout the protein, its function is not restricted to the SWI/SNF complex [2]. Mutations that result in loss of *ARID1A* have been shown to contribute to faster tumorigenesis, progression towards metastasis, and development of chemoresistance across several cancers including ovarian, colon, osteosarcoma, and esophageal cancer [2,3,4,5,6,7,8,9]. As an important tumor suppressor, it is essential to understand the mechanisms whereby *ARID1A* loss promotes various stages of cancer development. *ARID1A* is not only involved in various stages of cancer development but contributes to this through multiple hallmarks of cancer [10,11,12]. In this review, we explore the more recent evidence suggesting that *ARID1A* loss contributes to notable cancer hallmarks such as genomic instability, cell cycle regulation, EMT and dedifferentiation, immune evasion, and evading cell death across various cancer types. First, we present the correlation of pathogenic variants in the most impacted cancer types.

## 2. Clinical Impact

### 2.1. Prevalence of ARID1A Mutations Across Cancers

*ARID1A* mutations are often associated more with cancer disease progression, rather than disease onset. In a pan cancer analysis, which included 40,167 cancer patients representing endometrial cancer (*n* = 847), bladder cancer (*n* = 1329), esophageal cancer (*n* = 1448), ovarian cancer (*n* = 238), cancer of unknown primary (*n* = 188), skin cancer, non-melanoma (*n* = 217), cholangiocarcinoma (*n* = 36), salivary gland cancer (*n* = 116), colorectal adenocarcinoma (*n* = 127), ampullary cancer (*n* = 176), hepatocellular carcinoma (*n* = 396), melanoma (*n* = 1497), lung cancer (*n* = 3382), pancreatic cancer (*n* = 1352), colorectal cancer (*n* = 1234), cervical cancer (*n* = 297), glioma (*n* = 705), anal cancer (*n* = 33), breast cancer (*n* = 4572), salivary cancer (*n* = 212), head and neck cancer (*n* = 400), and thyroid cancer (*n* = 231), it was found that 6.2% of patients’ tumors had *ARID1A* mutations, with 40% being characterized as missense mutations [13]. This spans many tumor types including endometrial, bladder, gastric, liver, biliopancreatic cancer, ovarian cancer, and other unknown aggressive cancers [14]. The frequency of mutations can vary greatly between different tumor types. Previous literature has determined ovarian clear cell carcinoma was the most common cancer type found with an *ARID1A* mutation, with endometrioid cancer being the second [14,15]. One study found that in ovarian clear cell carcinoma, there was an *ARID1A* mutation in 46–57% of tumors [16]. Various studies have looked at *ARID1A*’s effect on different cancer types. For example, the presence of ARID1A aids in the regulation of hepatocellular carcinoma, the most common type of liver cancer [17]. A study in urothelial cancer found that there was a 16% discordance between primary and metastatic *ARID1A* mutations. The mutation was often present in the metastatic tumor and absent from the primary tumor, indicating that *ARID1A* plays a more critical role in metastatic transformation [18]. It is unclear in the study whether these discordant mutations were newly acquired during the transformation to become metastatic or if they were present and selected for because of the ability to enhance phenotypes necessary to become metastatic. To the researchers’ credit, they resampled discordant tumors to rule out if cancer heterogeneity was a factor, but additional sequencing was not able to find the mutant *ARID1A.* They also reevaluated the bioinformatic pipeline to look beyond the standard variant allele frequency and still could not identify the mutant ARID1A, which suggests the acquisition of the mutation during the process of becoming metastatic or being present at a very low frequency in the primary tumor.

The hallmarks of cancer are a framework to understand the complexity of cancer and cancer growth. These hallmarks developed over time into 14 categories. These hallmarks consist of sustaining proliferative signaling, evading growth suppressors, avoiding immune destruction, enabling replicative immortality, activating invasion and metastasis, inducing vasculature, resisting cell death, deregulating cellular metabolism, senescent cells, unlocking phenotypic plasticity, genome instability, epigenetic reprogramming, polymorphic microbiomes, and tumor-promoting inflammation. Our aim of this review was to study and synthesize the prior research on *ARID1A* in relation to the most overlapping hallmarks [10].

### 2.2. Overview of ARID1A Mutation Types: Frequency and Pathogenicity

Loss of function and loss of expression of *ARID1A* is a driving force behind the initiation and progression of cancers. However, not all mutations within the *ARID1A* gene are driver mutations, and interestingly, it has been observed that the loss of *ARID1A* results in a more unstable genome, which impacts the *ARID1A* gene itself, introducing variants that are either benign or of unknown significance. While it appears that *ARID1A* exhibits more frameshift and nonsense mutations associated with cancer, several missense mutations impact protein stability, as delineated in a 2025 study by Goutam et al. They took the five most common missense mutations in the DNA-binding domain of *ARID1A* and performed experimental and computational analyses to evaluate protein stability. The variant R1020S exhibited the highest destabilization of the protein. Computational modeling of the variants did not show concordance with the experimental data, which underlines how little we know about protein folding and intramolecular interactions [19,20]. We performed our own query of common mutational hotspots across the entire *ARID1A* gene using 550 samples with confirmed known *ARID1A* pathogenic variants on the COSMIC database (cancer.sanger.ac.uk) [20]. Table 1 lists the 6 most frequent sites for *ARID1A* mutations.

In a small sequencing study focused on 15 ovarian samples with matched germline blood sequencing, the researchers found that 40% of the samples harbored a pathogenic somatic variant in *ARID1A*. None of the variants identified were found in the DNA-binding domain. One patient exhibited 3 missense mutations in *ARID1A*, while the other 5 patients exhibited only one pathogenic variant of *ARID1A*, two of which were frameshift mutations and two that were nonsense mutations [21]. In a larger cohort study of ovarian clear cell carcinoma in a different ethnic background, Yang et al. found again that *ARID1A* was genetically altered in 64.3% of patients (27/42). The distribution of mutations was scattered across the gene, which supports the idea that multiple variants are capable of disrupting the function of the protein. Despite being a putative tumor suppressor, whole exome sequencing identified a majority of heterozygous variants that consisted of 21 frameshift, 11 nonsense, 7 missense, 3 in-frame variants and 3 splice site variants spread across the coding exons of *ARID1A*, thus confirming other studies that have shown that a majority of *ARID1A* variants are frameshift and nonsense mutations [22,23,24,25].

Nonsense mutations are typically more consequential than missense mutations and often reoccur at the same positions in cancer genes across patient cohorts. A pan-cancer study of the four genes most enriched for nonsense mutations (*TP53*, *CDKN2A*, *PTEN*, *ARID1A*) found their distribution to be non-random. However, the pattern for *ARID1A* was starkly different. For *TP53*, *CDKN2A*, and *PTEN* few hotspots contained the majority of the nonsense mutations (32–74%). In *ARID1A*, nonsense mutations were more interspersed with the hotspots only containing 10–17% of nonsense mutations depending on whether TCGA or COSMIC databases were used. The hotspot mutation sites in *ARID1A* were p. 1335, p. 1721–1722, and p. 1989 [26]. Two of these hotspot mutation sites were also identified in our own analysis of the six most common pathogenic variants in *ARID1A*. Regardless of if it is a nonsense or a missense, disruption of amino acids 1721 and 1989 appear to be hotspots and likely disruptors of the protein’s function.

In a study of 50 endometrial carcinomas evaluating the correlation of *ARID1A* mutations with RNA and protein expression, 22/50 samples were lacking *ARID1A* expression. The most frequently observed variants were classified as frameshift and nonsense (10/22). There were 6 samples with missense mutations, and the remaining samples did not have any variants in *ARID1A* but still resulted in no ARID1A protein expression, indicating that epigenetic mechanisms are important to consider as driver mechanisms of cancer progression [20]. In another larger study that included ovarian clear cell carcinoma and endometrioid carcinoma, it was also demonstrated that a majority of samples that exhibited *ARID1A* mutations had a correlative absence of protein staining; however, there were samples with no mutations and no protein expression, corroborating the previous study [27].

One detailed analysis of in-frame INDELS in *ARID1A* demonstrated that removing the nuclear export signal from the peptide prevented export from the nucleus, which resulted in increased proteasomal degradation of the protein in the nucleus. This suggests that the stability of ARID1A is subcellular location dependent and that it is less stable within the nucleus than in the cytoplasm [28]. Another study found that point mutations were enough to significantly increase tumor size in cells with limited ARID1A expression in the nucleus. Interestingly, they found that by inhibiting XPO1, an exportin with affinity for point-mutated ARID1A proteins, they were able to restore close to normal ARID1A function. This suggests that at some point, mutations may not cause a loss of function in the protein but rather induce protein degradation [29].

### 2.3. Prognostic Factors Associated with ARID1A Mutations

*ARID1A* mutations exhibit diverse prognostic significance across different types of cancer, though the data on this topic remains somewhat controversial. Most often, *ARID1A* loss or deletion is associated with a worse prognosis. In gallbladder cancer, ARID1A expression negatively correlated with prognosis and was identified as an independent factor for overall survival. Additionally, low ARID1A expression was associated with the worst survival outcomes when combined with high PD-L1 expression [30]. A subsequent study on gastric cancer supported this finding, demonstrating an inverse relationship between ARID1A and PD-L1 expression while linking ARID1A loss to an increased tumor mutation burden [31,32]. This, in turn, suggested a greater likelihood of success with immune checkpoint inhibitor (ICI) treatment. Five-year overall survival (OS) rates were reported as 68.8% in ARID1A-positive patients compared to 52.2% in ARID1A-negative patients. However, in oral squamous cell carcinoma, patients generally exhibited a better prognosis when *ARID1A* mutations were detected [33], contradicting previous studies. Additionally, *ARID1A* mutations have been linked to improved responses to ICIs in multiple cancer types. In one study, ICI therapy was associated with improved overall survival in patients with *ARID1A* mutations (28 months vs. 18 months, *p* = 0.0092) [13]. Regarding treatment strategies, mismatch repair (MMR) deficiencies and synthetic lethality approaches appear promising, as *ARID1A* mutations contribute to MMR defects. Synthetic lethality has been explored for improving therapeutic outcomes with PARP and ATR inhibitors [34]. Additionally, one study investigated metabolic and epigenetic targeting, revealing increased glutamine metabolism in *ARID1A*-deficient cells, which suggests the potential for GLS1 inhibitors such as CB-839 [32]. It is also worth noting that in some cancers, *ARID1A* loss has been associated with resistance to certain chemotherapies. This resistance to chemotherapies such as platinum-based treatments may be due to the dysregulation of DNA repair mechanisms or by the alterations in drug metabolism pathways [35]. The therapeutic implications of altering *ARID1A* for biomarker potential proved to be positive. In one study, they concluded that *ARID1A* alterations merit further exploration in their use as a novel biomarker correlating with better results with checkpoint blockade immunotherapy. They were particularly effective in the following cancer types: endometrial, gastric, and colorectal [36]. There is some controversy that bears mentioning in both early-stage and late-stage prognoses for patients who suffer loss of *ARID1A*. The prognostic impact varies with early vs. advanced cancers, displaying better survival rates for patients that experience early-stage *ARID1A* loss with endometrioid endometrial carcinoma patients. This same study demonstrated that the POLE mutations in 10% of the cases that possessed altered *ARID1A* may contribute to more favorable results [37]. While the co-occurrence of POLE mutations and ARID1A mutations is rare, the combined synergy of impaired DNA proofreading during replication coupled with genomic instability and double-strand DNA breaks has demonstrated enhanced immune recognition that has the potential for favorable outcomes when using immune checkpoint inhibitors.

## 3. Hallmarks of Cancer

### 3.1. ARID1A Effect on Genomic Instability

*ARID1A* is the most frequently mutated member of the SWI/SWF complex, and as such, *ARID1A* mutations have been associated with increased genomic instability [38]. In recent years, more evidence has highlighted the specific pleiotropic effects of *ARID1A* mutations on creating genomic instability. While correlative human data can highlight the predicted phenotypes when *ARID1A* is lost, this review will also emphasize some of the mechanistic studies performed in mice and other cancer models. Relying exclusively on one or the other has limitations and could introduce bias and skew our understanding of the mechanisms of *ARID1A*. All the hallmarks presented in this review are supported by both mechanistic animal studies and human clinical observations. One study found that mice with an *Arid1a* knockout in the liver resulted in increased micronuclei formation as well as significant upregulation of genes related to negative cell cycle regulation, such as *CDKN1A*, and DNA damage repair, such as *γH2AX* [38]. In a subsequent study, *ARID1A* mutations in gastric cancer patients also increased mutation rates in many other cancer-related genes compared to patients with normal *ARID1A* [39].

*ARID1A* is also found to be essential for MSH2 localization, a mismatch repair protein. In *ARID1A*-deficient cells, MSH2 has trouble localizing to chromatin, causing DNA access impairment and mutation accumulation [40]. Another study found ARID1A vital for recognizing and resolving R-loops, or RNA binding to DNA during transcription, causing displacement of DNA non-template strands, which increases genomic instability when left unchecked [41]. Since *ARID1A* has been found to maintain proper chromatin structuring, loss of *ARID1A* also contributes to increased chromatin relaxation at enhancer sites, damaging chromatin architecture and driving super-enhancer hyperactivation in oncogenes [37].

Low expression of *ARID1A* has been attributed to increased persistence of double-strand breaks and reduced chromatin repair. Transcriptional analysis in one study found that in mouse osteosarcoma models, genomic instability was the highest-rated hallmark in *Arid1a* knockout in mice compared to wild type [9]. Additionally, the same study found that out of 150 DNA damage repair genes in these models, *Dclre1c* was the only gene directly correlated with Arid1a loss and decreased survival. *Dclre1c* encodes for the protein Artemis, which is involved in non-homologous end joining to repair double-strand breaks. This paper thus identifies a primary candidate gene that ties *Arid1a* loss to genomic instability through impaired DNA repair [9]. Similarly in the study by Park et al. *ARID1A* demonstrated a direct correlation with *DCLRE1C* and further highlighted that loss of *ARID1A* makes cancer susceptible when DNA is further exacerbated using ionizing radiation and PARP inhibitors [42]. A subsequent study investigating the impact of ARID1A in double-strand break repairs specifically discovered that, generally, double-strand breaks persist longer in *ARID1A*-deficient cells, leading to increased chromosomal rearrangements [43].

Notably, in some cancer types, *ARID1A* mutations have been associated with greater genomic stability based on DNA copy number variations [44]. This paradox was reconciled by a study that found that intact ARID1A directly binds to the promoter of *STAG1* to activate its expression. STAG1, in turn, promotes telomere cohesion during mitosis to preserve proper chromosome segregation and thus genomic stability. When *ARID1A* is mutated, *STAG1* expression decreases, resulting in chromosomal aberrations that lead to apoptosis. Over time, although *ARID1A* loss is causing apoptosis induced by genomic instability, some populations of *ARID1A*-deficient cells stochastically manage to avoid gross chromosomal aberrations and thus survive and continue to grow. Sampling tumors where this takes place reveals cells that appear genomically stable overall [44].

The genomic instability induced by ARID1A loss has putative mechanisms in conferring resistance to therapies. Through mechanisms of genomic instability, heterogeneity within a tumor increases. The presence of multiple genetic subclones of a tumor provides innate mechanisms of chemoresistance. Layer on top of the genetic heterogeneity that complex epigenetic regulation of gene expression by remodeling chromatin, tumors with ARID1A mutations could be hard to achieve durable responses to targeted and generic chemotherapy [8].

### 3.2. ARID1A in the Regulation of the Cell Cycle

Evidence that *ARID1A* functions as a tumor suppressor gene in the cell cycle by blocking uncontrolled growth is evident in the literature from studies where *ARID1A* is knocked down. For example, *ARID1A* loss disrupts the G2/M transition, which can cause cells with DNA damage to bypass G2/M checkpoints and enter mitosis prematurely. In renal cell carcinoma and endometrial cancer cells, *ARID1A* knockout increased the proportion of cells in the G2/M phase, showing a loss of proper checkpoint control [45]. Loss of ARID1A leads to reduced levels of CHK1, CHK2, and phosphorylated p53, affecting the DNA damage response and checkpoint regulation [46]. This disruption is significant because ARID1A works in concert with p53 to induce the expression of p21, a key cyclin-dependent kinase (CDK) inhibitor that halts cell cycle progression. When ARID1A is lost, p21 expression diminishes, resulting in unchecked CDK activity, accelerated cell cycle progression, and uncontrolled cell proliferation [3,47]. Moreover, knocking down p21 in ARID1A-expressing cells reverses growth suppression. This shows that p21 is a key mediator of ARID1A’s tumor-suppressive function.

Additionally, other studies show that the downregulation of *ARID1A* affects the expression levels of important cell cycle-governing genes. ARID1A is part of the SWI/SNF chromatin remodeling complex, which regulates gene accessibility. Thus, ARID1A loss leads to global chromatin condensation which in turn affects the transcription levels of important genes monitoring the cell cycle, such as *P52*, *PARP1*, *DDB1*, and *SFPQ* [48]. Another study found that the downregulation of *ARID1A* affects the expression level of the E2F-responsive genes *cdc2* and *c-Myc*. These genes encode transcription factors that recruit RNA polymerase to transcribe genes that promote cell growth. ARID1A normally suppresses E2F-responsive genes, preventing excessive proliferation. Without ARID1A, however, these genes are overactivated, driving unchecked cell cycle progression [49].

Downregulation of *ARID1A* not only promotes cancer cell proliferation but also contributes to metastatic potential and increased genomic instability. For example, in renal cell carcinoma, *ARID1A*-deficient cells showed decreased apoptosis and increased TGF-β1 (Transforming Growth Factor beta 1) protein expression, which promoted metastasis and epithelial–mesenchymal transition [49]. In stem cell studies, ARID1A was shown to regulate the transition from proliferation to differentiation. Knockout in mouse incisors showed an increase in proliferative transit-amplifying cells (TACs), which caused the cells to fail to exit the mitotic cycle and continue proliferating [50]. Additionally, the knockout of *ARID1A* in endometrial cancer cells decreased pro-apoptotic proteins (caspase 7, caspase 9, and p21), leading to increased survival and proliferation of damaged cells [45]. *ARID1A* deficiency can also lead to mismatch repair defects, replication stress, and genomic instability, making *ARID1A*-mutant cancers vulnerable to specific therapies, including PARP inhibitors, EZH2 inhibitors, BET inhibitors, and ATR inhibitors [25]. This suggests that treatments targeting these vulnerabilities could be useful in counteracting low *ARID1A* expression and its effect on the cell cycle in cancer cells.

### 3.3. ARID1A Regulation of EMT and Cell Differentiation

The Epithelial–Mesenchymal Transition (EMT) refers to the biological process where a polarized epithelial cell, which typically adheres to the basement membrane through its basal surface, can undergo chemical processes to adopt a mesenchymal cell phenotype. Increasing evidence suggests that *ARID1A* directly regulates EMT and cell differentiation across several tissues and cancer types. Independent studies on pancreatic ductal adenocarcinomas, renal cell carcinomas, colorectal cancer cells, and breast cancer cells exhibited mutations to the *ARID1A* gene which caused an increase in mesenchymal markers N-Cadherin, TGF-ß, and vimentin as well as a decrease in epithelial markers E-cadherin and zonula occludens-1 (ZO-1) [45,51,52,53]. In contrast, restoring *ARID1A* expression led to an increase in epithelial markers and a decrease in mesenchymal markers.

Gene enrichment analysis on *ARID1A* deficient cells reveals activation of several pathways involved in EMT, cell motility, cell migration, regulation of immune response, and chemotaxis. These factors are commonly linked to a tumor’s ability to metastasize and suggest that the loss of *ARID1A* increases EMT and enhances metastatic potential [54].

In a functional characterization paper, the authors investigated the effects of overexpressing and knocking down *ARID1A* in ovarian cancer cell lines and xenograft models. *ARID1A* overexpression resulted in a more differentiated ovarian cancer cell line and upon knockdown, the same cancer cell lines started expressing cancer stem cell markers: NANOG, OCT3/4, and SOX2. The authors further demonstrated that *ARID1A* expression is positively regulated by the HIPPO pathway, and as a result, it reinforces HIPPO signaling, forming a tumor-suppressive feedback loop. Together, they inhibit the expression of cancer stem cell markers and the phenotypes that are associated with cancer stem cells. The downstream effector of the HIPPO pathway, TAZ, is expressed in ovarian cancers without *ARID1A* indicating that TAZ inhibitors could be used to induce synthetic lethality or an agonist that promotes *ARID1A* expression. Additionally, researchers created nutrient-deprived dual-layer membrane assays where they placed *ARID1A*-deficient epithelial cells at the top layer. Below it, they placed a nutrient-enriched membrane. They demonstrated that the epithelial cells were able to transition into mesenchymal cells and migrate to the nutrient-rich environments on the bottom layer, providing evidence for the EMT-mediated migration potential [55].

Similarly, in a study of head and neck cancer, cancer cells expressed high levels of NANOG, OCT4, SOX2 and EpCAM when ARID1A was absent. This downregulation of ARID1A was inversely correlated to the oncogenic microRNA, miR-31. This demonstrates that loss of function mutations of the ARID1A gene itself is not the only way to achieve the cancer hallmark of cellular plasticity [56].

Interestingly, *ARID1A*’s role in cell fate seems to be context-specific. In a study on the role of *ARID1A* in developing cardiogenic stem cells and neurogenic stem cells, the authors concluded that *ARID1A* had opposite roles. In cardiogenic differentiation, *ARID1A* is required to promote chromatin accessibility in heart-specific genes. During neurogenic differentiation, however, *ARID1A* appears to suppress the activity of neuronal genes by potentially interacting with the transcriptional repressor REST/NRSF [57]. These non-cancer studies underline the context-specific role of ARID1A to impact gene expression that influences the cell fate or stemness of a given cancer cell.

Finally, in highly invasive and metastatic breast cancer cells, the transfection of *ARID1A* into the cells caused an increase in epithelial marker E-cadherin and a decrease in mesenchymal markers vimentin and N-Cadherin. When combined with the chemotherapy agent Fluorocil (5-FU), epithelial markers increased further. This provides some evidence suggests that, in cells already deficient in *ARID1A*, transfecting the cells with *ARID1A* has the potential to increase their epithelial properties and potentially decrease the metastatic potential of the cancerous cells [51].

### 3.4. Immune Evasion and Modulation in ARID1A-Deficient Tumors

Low ARID1A expression is associated with altered expression of various proteins involved in immune evasion. One of the most common proteins expressed on the outside of tumor cells to evade immune detection when *ARID1A* is low is PD-L1, which is a protein that binds to the PD-1 protein on T cells and signals them to stop attacking the cancer cell. This inverse relationship of ARID1A and PD-L1 is seen in gallbladder cancer, lung cancer, and endometrial cancer [30,37,58,59]. In gastric cancer, it was shown that when *ARID1A* is lost an increase in PD-L1 protein expression is seen; this finding was attributed to an uptick in activity of the P13K-AKT signaling pathway which promotes growth and cell division [60]. High PD-L1 expression due to the downregulation of the *ARID1A* gene is also seen in gallbladder cancer, where CD8+ T cells are inactivated as a result [45]. Higher PD-L1 expressions are also seen in colorectal cancer due to low *ARID1A* expression. Interestingly, however, the same study found that low expression of *ARID1A* also brought more cytotoxic T cells and monocytic lineage immune cells to the tumor microenvironment [61].

Another protein that has been correlated with immune evasion due to the knockdown of *ARID1A* is the CD47 protein. The CD47 protein is expressed on the surface of a cell membrane and is a part of the immunoglobulin family of proteins. When a macrophage binds to the CD47 protein, it produces a signal through the macrophage’s own SIRPα protein receptor used to bind to CD47 and tells it not to engulf it through phagocytosis. This allows the tumor cell to evade immune detection and destruction. It has been shown that when ARID1A expression is low in gastric cancer, the CD47 protein is elevated so that the immune response by M1 macrophages can be negated via increased T regulatory cells and M2 macrophages [62].

One area of interest in low *ARID1A*-expressing cancer cells is the impairment of its mismatch repair function. When the mismatch repair function in the cancer cell is not working properly due to a lack of the *ARID1A* gene, any mutation that occurs may create a neoantigen on the surface of the cancer cell that can confer immune cell recognition. However, the concomitant upregulation of PD-L1 can negate the stimulation of immune cells due to the presence of these neoantigens resulting in immune cell evasion capabilities [40,61].

*ARID1A* low expression is also shown to increase the number of myeloid-derived suppressor cells (MDSCs) via an increased activation of the NF-κB signaling pathway to produce more CXCL2 and CXCL3 chemokines to recruit MDSCs to the tumor microenvironment. MDSCs suppress immune cell activity and benefit the tumor’s longevity with their presence as a result [63].

Notably, low expression of *ARID1A* is also shown to increase the immune response in lung cancer. One specific example of this is the inhibition of autophagy within the tumor cell itself, leading to an increase in interferon gamma signals being produced via increased activation of the PI3K/Akt/mTOR pathway. It is hypothesized that *ARID1A* inhibits the activation of the EGFR/PI3K/Akt/mTOR pathway through its ability to reposition nucleosomes that condense the chromatin around these genes. Without *ARID1A*, the chromatin opens and allows for active transcription. Interferon gamma serves as a chemoattractant to leukocytes facilitating the invasion into the tumor microenvironment. The increased immune response in patients with low *ARID1A* expression has proven effective for better progression-free survival when treatments include ICI’s [58].

### 3.5. ARID1A Loss and the Impact on Apoptosis

Recent research published regarding ARID1A’s impact on apoptotic pathways has been more limited. The studies that have been conducted largely focus on ways that ARID1A expression or loss leads to the downregulation or upregulation of genes associated with different apoptotic pathways.

One study found that the knockout of *ARID1A* led to a decrease in apoptosis. This study looked primarily at *ARID1A*’s effect on retinal ganglion cells (RGCs). After performing gene set enrichment analysis (GSEA), it was indicated that genes related to apoptosis were downregulated in *ARID1A* knockout mice RGCs compared to the wild-type RGCs mice. KEGG pathway analysis was also performed on the differentially expressed genes in *ARID1A* knockout RGCs, showing a correlation with pathways related to apoptosis. Through *ARID1A* deletion, these findings demonstrate that apoptosis programs may be repressed, leading to improved survivability. In particular, they saw that *ARID1A* is positively correlated with other genes associated with neuronal survival, such as *STAT1*, and may impact JAK-STAT pathways [64].

Another study measured cell death between ARID1A-expressing and -deficient tumor xenografts when treated with a combination of a PARP inhibitor and ionizing radiation. These researchers found that mice with ARID1A-expressing tumors, PARP inhibition did not have any additional anti-tumor benefits that irradiation did not have. In mice with ARID1A-deficient tumors, the combination of both therapies demonstrated greater efficacy compared to either treatment alone. Additionally, IHC staining revealed there were significantly elevated levels of double-strand breaks (DSBs) and cell death in the ARID1A-deficient tumors compared to the ARID1A-expressing tumors following the combination treatment. This difference is hypothesized to be due to ARID1A’s role in DNA repair pathways [42].

Lastly, a study found that HDAC6 (Histone deacetylase 6) was upregulated in ovarian cancers with *ARID1A* mutations. This enzyme deacetylates Lys-120 on p53 proteins, negatively impacting the pro-apoptotic features of p53. They concluded that *ARID1A* mutation, by the upregulation of HDAC6, inactivates the apoptotic function of p53 [65]. This observation corroborates a study in colon cancer that demonstrated that TP53 gene mutations and ARID1A gene mutations were mutually exclusive [61].

## 4. Conclusions

The tumor suppressor gene, *ARID1A*, is a significant driver of mutation across various cancer types, but most prominently in ovarian cancer types. While the canonical role of ARID1A is to facilitate chromatin remodeling as a member of the SWI/SNF complex, this epigenetic regulation can lead to pleiotropic effects as it broadly impacts gene expression. In addition to its canonical role, ARID1A is known to have additional binding partners, thus expanding its impact when it is missing. This underlines the significant involvement across multiple cancer hallmarks. Many of its roles remain poorly understood and appear to be context-dependent. For example, although ARID1A is a tumor suppressor, certain cancer hallmarks are amplified following the loss of heterozygosity. The observation that biallelic loss is often absent, despite these effects, suggests that epigenetic mechanisms may contribute to tumorigenesis even with the loss of a single allele. Because of ARID1A’s canonical role in chromatin remodeling, it is no surprise that genomic instability is the most commonly featured cancer hallmark. The interplay of enhanced genomic instability resulting in increased neo-antigen expression explains the clinical observations that ARID1A loss portends a more favorable outcome in patients when treated with immune checkpoint inhibitors. As we understand more about the molecular mechanisms of ARID1A in different cancers, we can exploit the vulnerabilities therapeutically. This will become more apparent in cases of synthetic lethality where the loss of ARID1A makes cancer cells more susceptible to therapies such as ATM and PARP inhibitors. As more pathogenic variants of ARID1A are identified and more targeted cancer therapies are developed there will be molecular profiles involving ARID1A that guide clinical decisions that result in better outcomes for patients with cancer.

## Figures and Tables

**Table 1:** Top Six Most Common Somatic Pathogenic *ARID1A* Variants from COSMIC Database.

AA Position	R1989 *	R1276 *	R1721 *	R693 *	G2087R	R1335 *
cDNA Position	5965C>T	3826C>T	5161C>T	2077C>T	6259G>A	4003C>T
Total number of mutations out of 550 samples	65	38	31	31	24	24

*, denotes a change in the amino acid sequence to anything other than the original.

## Abbreviations

The following abbreviations are used in this manuscript:

CDK	Cyclin-dependent Kinase
COSMIC	Catalog Of Somatic Mutations in Cancer
DSB	Double-Strand Break
5-FU	5-Fluorouracil
GSEA	Gene Set Enrichment Analysis
HDAC	Histone Deacetylase
EMT	Epithelial–Mesenchymal Transition
ICI	Immune Checkpoint Inhibitor
INDEL	Insertion-deletion
MDSC	Myeloid Derived Suppressor Cell
MMR	Mismatch Repair
OS	Overall Survival
RGC	Retinal Ganglion Cell
TAC	Transient Amplifying Cells
ZO-1	Zonula Occludens-1
